# Inadequate Ultrasound Examination in Hepatocellular Carcinoma Surveillance: A Systematic Review and Meta-Analysis

**DOI:** 10.3390/jcm10163535

**Published:** 2021-08-12

**Authors:** Seung Baek Hong, Dong Hwan Kim, Sang Hyun Choi, So Yeon Kim, Ji Sung Lee, Nam Kyung Lee, Joon-Il Choi

**Affiliations:** 1 Department of Radiology, Biomedical Research Institute, Pusan National University Hospital, Pusan National University School of Medicine, 179 Gudeok-ro, Seo-gu, Busan 49241, Korea; cinematiclife7@pusan.ac.kr (S.B.H.); leenk@pusan.ac.kr (N.K.L.); 2 Department of Radiology, Seoul St. Mary’s Hospital, College of Medicine, The Catholic University of Korea, 222 Banpo-daero, Seocho-gu, Seoul 06591, Korea; kimdh@catholic.ac.kr (D.H.K.); dumky@catholic.ac.kr (J.-I.C.); 3 Department of Radiology and Research Institute of Radiology, University of Ulsan College of Medicine, Asan Medical Center, 88 Olympic-Ro 43-Gil, Songpa-Gu, Seoul 05505, Korea; sykimrad@amc.seoul.kr; 4 Department of Clinical Epidemiology and Biostatistics, University of Ulsan College of Medicine, Asan Medical Center, 88 Olympic-Ro 43-Gil, Songpa-Gu, Seoul 05505, Korea; jisung.lee@amc.seoul.kr

**Keywords:** hepatocellular carcinoma, surveillance, ultrasound, nonalcoholic steatohepatitis, body mass index, systematic review, meta-analysis

## Abstract

We aimed to systematically evaluate the incidence of inadequate US in hepatocellular carcinoma (HCC) surveillance and determine the risk factors. Original studies reporting the incidence or risk factors for inadequate US were identified in MEDLINE, EMBASE, and the Cochrane database. The pooled incidence of inadequate US was calculated using a random effects model, and subgroup analyses were performed. The pooled odds ratio (OR) was calculated for each risk factor for inadequate US. Six eligible articles were identified from 756 screened articles (4250 patients). The pooled incidence of inadequate US was 21.5%. Significantly higher rates of inadequate US were noted in studies including patients with and without hepatic observations compared with those evaluating only patients with hepatic observations (23.2% vs. 18.8%), studies using US alone compared with US plus alpha-fetoprotein (28.0% vs. 20.8%), and those using pathology and imaging as a reference standard compared with imaging only (23.2% vs. 17.9%). Nonalcoholic steatohepatitis (OR = 2.3 (1.07–4.84)), Child–Pugh B cirrhosis (OR = 2.2 (1.10–4.37)), and high body mass index (OR = 2.2 (1.12–4.24)) were significant risk factors for inadequate US (*p* ≤ 0.04). In patients at risk of HCC, 21.5% of US surveillance was inadequate. An alternative surveillance modality might be considered in patients with risk factors.

## 1. Introduction

Hepatocellular carcinoma (HCC) is the fifth most common cancer in the world and the third most frequent cause of cancer-related mortality [1,2]. The prognosis for patients with HCC is quite poor, with an overall 5-year survival rate below 20%, but patients who are diagnosed at an early stage are responsive to curative treatment, and 5-year survival rates of up to 70% can be achieved [3,4]. Given the fact that patients with early-stage HCC have a better prognosis than those with advanced HCC, and more than 80% of HCC cases are among patients at risk [5], regular surveillance to detect early-stage HCC in at-risk populations is clinically important.

The American Association for the Study of Liver Disease (AASLD) and the European Association for the Study of the Liver (EASL) recommend ultrasound (US) every 6 months as a standard surveillance modality [6,7]. US has many advantages, such as being an inexpensive and noninvasive method without any risk or radiation exposure for patients compared with computed tomography (CT) or magnetic resonance imaging (MRI) [8]. In addition, contrast-enhanced US can be useful to characterize dynamic enhancement patterns with a high predictive value for diagnosing HCC [9]. Although US surveillance can lead to the early diagnosis of HCC and improved survival [10,11], the sensitivity of US for detecting early-stage HCC is suboptimal, with a value of 47% being found in a recent meta-analysis [12]. In this context, the AASLD and EASL guidelines updated in 2018 suggest that alternative surveillance imaging modalities including CT or MRI may be needed in selected patients with a high likelihood of having an inadequate US examination [6,7].

Given the high diagnostic performance of CT or MRI for diagnosing small HCC (<2 cm; 68% sensitivity and 98% specificity for CT, 76% sensitivity and 96% specificity for MRI), and the ability to provide proper management based on accurate HCC diagnosis and staging [13,14,15], clinical attention to CT or MRI in HCC surveillance is increasing. In particular, recent studies have reported the clinical usefulness of MRI in HCC surveillance, including the use of abbreviated MRI protocols [16,17,18]. However, performing surveillance MRI in all at-risk patients may not be acceptable because of the high cost and limited radiologic capacity for MRI [19]. Therefore, understanding the reasons for US surveillance failure and identifying those patients for whom US is likely to be inadequate for evaluating HCC is important for improving the effectiveness of surveillance.

Some studies have reported on the incidence or risk factors of inadequate US examination [20,21,22,23,24,25], but they were retrospective single-center studies with limited generalizability to clinical practice. In addition, the reported results vary between studies, with one study reporting that male patients, cirrhosis, nonalcoholic steatohepatitis (NASH), and spleen size were significant risk factors [21], whereas another study reported that obese patients, those with Child–Pugh B or C cirrhosis, and those with alcohol- or NASH-related cirrhosis had a significantly higher risk of inadequate US examination [25].

Therefore, we aimed to systematically evaluate the incidence of inadequate US examinations and determine the risk factors for them.

## 2. Materials and Methods

This study followed the Meta-analysis of Observational Studies in Epidemiology (MOOSE) [26] and Preferred Reporting Items for Systematic Reviews and Meta-Analyses (PRISMA) [27] guidelines for conducting and reporting meta-analyses.

### 2.1. Literature Search Strategy

A comprehensive search of MEDLINE, EMBASE, and the Cochrane database was conducted. The search query was designed to perform a literature search with maximum sensitivity. A manual evaluation of the identified articles was then performed to narrow down the number of relevant articles. The search terms included “hepatocellular carcinoma”, “ultrasound”, “surveillance”, and “screen”. Supplementary Table S1 lists the search terms in detail. The literature search was updated until 1 September 2020, with no start date limit. The search was limited to human patients and English-language articles.

### 2.2. Eligibility Criteria

After the removal of duplicate articles, the identified articles were reviewed to determine their eligibility according to the following criteria: (1) patients: treatment-naïve patients at risk for HCC; (2) index test: US for HCC surveillance; (3) comparison: no comparison; (4) outcome: incidence and risk factors for inadequate US examination; and (5) study design: observational studies (prospective or retrospective) and clinical trials. Patients at risk for HCC included patients with cirrhosis or chronic liver disease. Surveillance was defined as the repeated use of the test at a regular interval over time for the detection of previously undiagnosed lesions [12], and studies performing evaluations for diagnostic purposes instead of surveillance were not included in this analysis. Inadequate US examination was defined as insufficient visualization of the entire liver or diaphragm, and limitations such as moderate to severe heterogeneous liver parenchyma, shadowing, or beam attenuation, and non-detection of a lesion on US that was found on another imaging modality [21,24,25]. Studies were excluded according to the following criteria: (1) case reports, letters, scientific abstracts, animal studies, review articles, and meta-analyses; (2) studies with overlapping data and patient cohorts; and (3) studies not within the field of interest. Two reviewers (S.B.H. and D.H.K.) independently performed the first screen of the retrieved articles according to their titles and abstracts with blinded information about authors and institutions, and then performed full-text reviews of the articles identified as potentially eligible. Disagreements between the two independent reviewers were resolved at a consensus meeting with a third reviewer (S.H.C.).

### 2.3. Data Extraction

The following data were extracted from eligible articles using a predefined data form: (1) study characteristics (author, study location, year of publication, and study design); (2) study population characteristics (patient numbers, age, sex, underlying etiology, number of patients with HCC, and number of patients with cirrhosis); (3) US examination techniques (US machine and sonographer experience); (4) details of US surveillance (the use of alpha-fetoprotein, US surveillance interval, and follow-up periods); (5) reference standard for HCC; and (6) study outcomes (incidence and risk factors for inadequate US examination). The number of inadequate US examinations was extracted from each individual study, and to assess the risk factors for inadequate US examination, the odds ratio (OR) of each risk factor and the corresponding 95% confidence interval (CI) was also extracted from each study. When not explicitly reported, ORs were manually extracted from the text and tables. Two reviewers (S.B.H. and D.H.K.) independently performed the data extraction. Cases showing discrepancies were discussed in a consensus meeting with a third reviewer (S.H.C.).

### 2.4. Assessment of Study Quality

Two independent reviewers assessed the quality of each individual study using the Newcastle–Ottawa Scale (NOS) [28]. The NOS has three domains, including the selection of the study individuals, the comparability of the study groups, and ascertainment of the study outcome, with a maximum possible score of nine. Studies with NOS scores < 7 were considered to have a high risk of bias and those with scores ≥ 7 had a low risk of bias.

### 2.5. Data Synthesis and Statistical Analysis

For the available literature, the incidence of inadequate US examinations was calculated for each individual study by dividing the number of inadequate US examinations by the total number of US surveillance examinations. To determine the pooled incidence of inadequate US examination, the inverse variance method was used to calculate weights, and the percentages and their 95% CIs were obtained using a restricted maximum-likelihood estimation random effects model. Study heterogeneity was assessed using the Higgins *I*^2^ statistic, with an *I*^2^ > 50% being considered to indicate substantial heterogeneity. Subgroup analyses were performed using meta-regression to evaluate the following covariates: (1) surveillance cohort (total cohort vs. patients with hepatic observations); (2) geographic differences (North America vs. others); (3) proportion of patients with cirrhosis (all with cirrhosis vs. not all with cirrhosis); (4) surveillance test (US alone vs. US plus AFP); (5) US system (multiple vs. single); and (6) reference standard (pathologic and imaging diagnosis vs. imaging diagnosis only).

The OR of each risk factor for inadequate US examination was extracted from each study. The OR is a ratio of the odds of inadequate US examination given exposure to the risk factor to the odds of inadequate US examination given a lack of exposure to the risk factor. A random effects model was used to calculate the meta-analytic pooled OR and its 95% CI for each risk factor for inadequate US examination.

Publication bias was assessed using funnel plots, plotting the effect size and the measure of the precision of the effect size. Visual assessment was complemented with Egger’s test for funnel symmetry.

All statistical analyses were performed using R version 3.3.2 (The R Foundation for Statistical Computing, Vienna, Austria) with the “meta” package, with *p* < 0.05 being considered statistically significant.

## 3. Results

### 3.1. Literature Search

**Figure 1:**
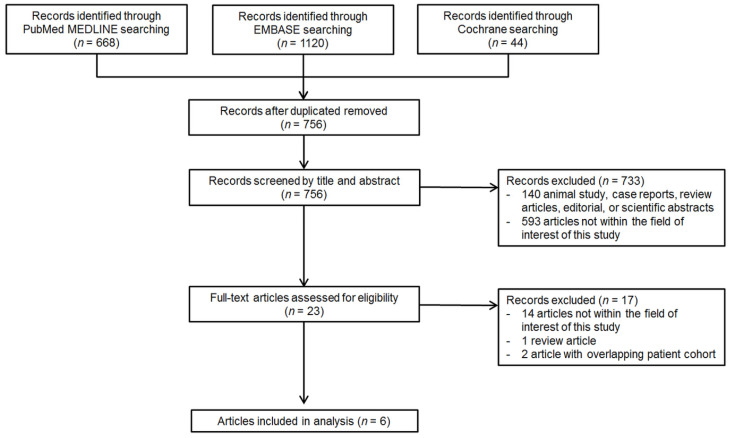
PRISMA flow diagram of article selection.

A total of 756 articles were screened after the removal of duplicates. Of these, 733 articles were excluded based on the title and abstract, and an additional 17 articles were excluded after full text review. Finally, six eligible articles reporting both the incidence of inadequate US examination and the risk factors for inadequate US examination were included. A flow diagram of article selection is presented in Figure 1.

### 3.2. Study Characteristics

The characteristics of the eligible articles are presented in Table 1. All six studies were retrospective studies [20,21,22,23,24,25]. Four studies were from Western countries [21,22,24,25], and two were from Eastern countries [20,23]. Three studies included both patients with and without hepatic observations in the US surveillance [21,23,25], whereas the other three studies only included patients with hepatic observations [20,22,24]. The most common underlying liver disease was hepatitis C in three studies [22,24,25], and hepatitis B in the other three studies [20,21,23]. Five studies performed US surveillance with AFP [20,21,22,24,25], whereas one performed US alone [23]. Regarding the US technique, two studies used multiple US systems [21,25], one used a single US system [23], and the others were unclear as to whether single or multiple US systems were used [20,22,24]. Five studies performed US surveillance at 6-month intervals [20,21,22,23,25], but one study did not clarify the surveillance interval [24]. Both pathologic and imaging diagnoses were used in four studies as a reference standard for HCC [20,21,22,23], whereas one study only used imaging diagnosis [24].

### 3.3. Study Quality

Of the six included studies, four were considered at a low risk of bias [20,21,23,25] (NOS score ≥ 7; Supplementary Table S2) and two were considered at a high risk of bias [22,24]. In the selection of the study individuals’ domain, all studies had a representative inadequate US cohort, and included an adequate US cohort from the same community as the inadequate US cohort. Regarding the comparability of the study groups, one study did not compare results between the adequate and inadequate US groups [22]. In the study outcome domain, two studies were unclear on how they evaluated and determined the study outcomes, i.e., the use of independent blind assessment [22,24]. In addition, the duration of follow-up was available for two studies [21,23], but not for the other four [20,22,24,25].

### 3.4. Incidence of and Risk Factors for Inadequate US Examination

**Figure 2:**
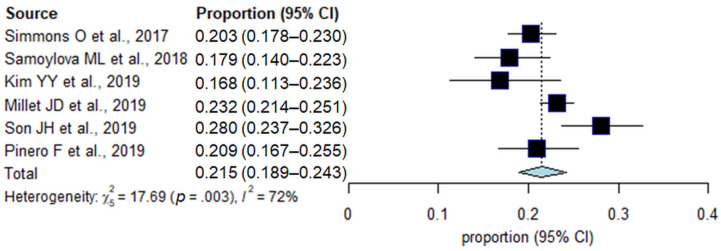
Forest plots of the pooled incidence of inadequate US examination.

The incidences of inadequate US examination in each study are summarized in Figure 2. In a total of 4250 patients in six studies, the pooled incidence of inadequate US examination was 21.5% (95% CI, 18.9–24.3%; *I*^2^ = 72%). In subgroup analyses, the surveillance cohort, surveillance test, and reference standard were significantly associated with study heterogeneity (*p* ≤ 0.04; Table 2). In studies including both patients with and without hepatic observations in the surveillance cohort, the incidence of inadequate US examination was 23.2%, which was significantly higher than that in studies including only patients with hepatic observations (18.8%, *p* = 0.03). In addition, a higher incidence of inadequate US examination was shown in studies that used US alone compared with those that used US plus AFP (28.0% vs. 20.8%, *p* = 0.008), and in those that used pathologic and imaging diagnoses as a reference standard compared with those that used imaging diagnosis only (23.2% vs. 17.9%, *p* = 0.02).

**Figure 3:**
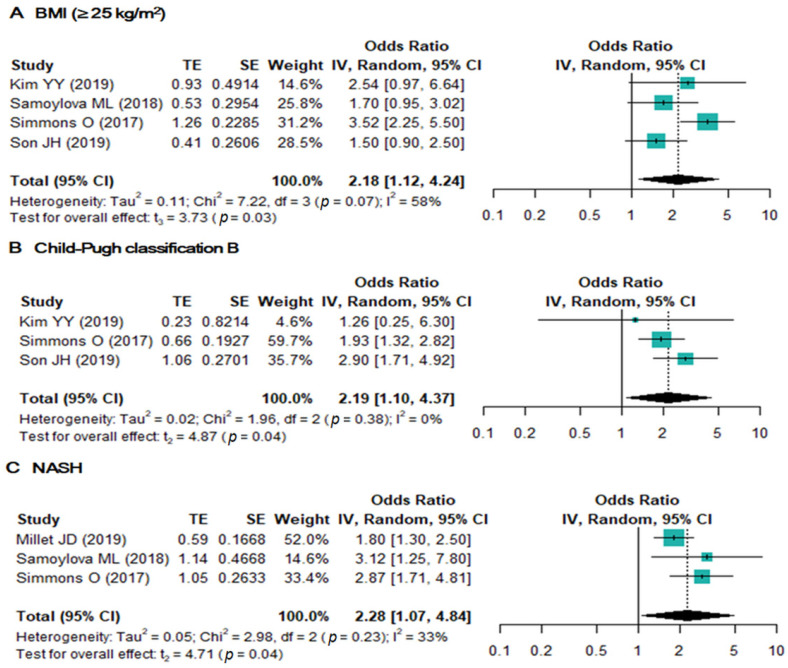
Forest plots of the odds ratios of BMI (≥25 kg/m^2^) (**A**), Child–Pugh classification B (**B**), and NASH (≥100 ng/mL) (**C**).

A total of nine risk factors in five studies were available for analysis of inadequate US examination (Table 3). Of these nine risk factors, high body mass index (BMI ≥ 25 kg/m2), Child–Pugh B cirrhosis, and NASH were significantly associated with inadequate US examination (Figure 3). In addition, NASH demonstrated the highest pooled OR (2.3 (95% CI, 1.07–4.84); *I*^2^ = 33%), followed by Child–Pugh Classification B (2.2 (95% CI, 1.10–4.37); *I*^2^ = 0%) and high BMI (2.2 (95% CI, 1.12–4.24); *I*^2^ = 58%). The other six potential risk factors showed no statistically significant associations (Supplementary Figure S1).

No significant publication bias was noted across the studies (*p* = 0.418, Supplementary Figure S2).

## 4. Discussion

Our meta-analysis found that inadequate US examinations occurred with an incidence of 21.5% (95% CI, 19.1–24.3%) in HCC surveillance. This result is in line with the proportion of HCC diagnosed beyond the Milan criteria in the Hepatitis C Antiviral Long-term Treatment Against Cirrhosis (HALT-C) trial population (27.7%) [29]. Considering both the non-negligible incidence of inadequate US examination and the proportion of HCC diagnosed beyond the early stage in US surveillance, alternative surveillance imaging modalities including CT or MRI should be considered to improve HCC surveillance and lead to proper management according to each patient’s risk for HCC.

In the subgroup analyses, studies including patients with and without hepatic observations had a significantly higher incidence of inadequate US examination than those including only patients with hepatic observations. Because studies including only patients with hepatic observations did not address the quality of US surveillance in patients without hepatic observations, these results have limited generalizability to clinical practice. In other words, the 23.2% inadequate US rate in studies including both patients with and without hepatic observations may be regarded as a conservative estimate of the incidence of inadequate US examination. In addition, studies that used pathologic and imaging diagnosis as a reference standard had a significantly higher incidence of inadequate US examination than those that used imaging diagnosis only. Because imaging diagnosis, including multiphasic CT and MRI, may not be perfect, i.e., a 66% sensitivity and 92% specificity for CT, and 82% sensitivity and 91% specificity for MRI [13], and the performance of CT and MRI is poor for HCC < 2 cm [13] which is the major target in HCC surveillance, the incidence rate of 23.2% in studies that used both pathologic and imaging diagnosis as a reference standard should be a more reliable estimate.

Child–Pugh B cirrhosis was found to be a significant risk factor for inadequate US examination. Although there was not a significant difference in the incidence of inadequate US examination between studies exclusively enrolling patients with cirrhosis and those not exclusively enrolling patients with cirrhosis, it was not possible to determine whether or not cirrhosis was a significant risk factor for inadequate US examination because patients with cirrhosis still formed part of the cohort in the studies that did not exclusively enroll patients with cirrhosis [21]. As liver fibrosis progresses to cirrhosis, the number of regenerated nodules consisting of fibrous septa increases and the appearance of liver parenchyma becomes inherently distorted, making it harder to detect early HCC [20,30,31]. In addition, a severely shrunken liver in Child–Pugh B or C cirrhosis is also more difficult to visualize, as most of the liver is retracted under the rib cage, even at deep inspiration [25]. For context, in several previous prospective studies exclusively enrolling patients with cirrhosis, 19–31% of HCC was diagnosed beyond the early stage with US surveillance [32,33,34].

Our study found that high BMI and NASH were significantly associated with inadequate US examination. As the US beam is more likely to be attenuated by thick subcutaneous fat, the quality of US images of the entire liver may be diminished in patients with high BMI [35]. Similarly, steatohepatitis can exacerbate attenuation of the US pulse and result in poor visualization of deep structures [36]. Although Son et al. reported that moderate to severe hepatic steatosis was a significant risk factor for a poor US visualization score [23], our study could not evaluate whether simple hepatic steatosis without inflammation was significantly associated with inadequate US examination because of a lack of eligible studies. Further study is needed to determine the association between simple hepatic steatosis and inadequate US examination in HCC surveillance.

Surveillance with US plus AFP showed a significantly lower incidence of inadequate US examination compared with US alone. This result is similar to previously reported findings that adding AFP to US surveillance is associated with significantly improved sensitivity [12]. However, given the suboptimal performance of surveillance with US plus AFP for detecting early-stage HCC, MRI including abbreviated MRI could be considered as an alternative surveillance modality in patients at risk for HCC. In particular, hepatobiliary contrast (HBA)-enhanced abbreviated MRI is promising. Because HBA is taken up by hepatocytes by means of hepatocyte-specific organic anion-transporter protein (OATPs) and OATP expression decreases during carcinogenesis before complete neoarterialization, hepatobiliary-phase imaging may allow the detection of additional lesions such as small or early-stage HCCs that are not visible on images from any other sequences [37]. In addition, the degree of OATP1B1/3 expression correlates inversely with HCC tumor grade and the presence of biliary phenotypic markers, such as biliary-type keratin 7 and keratin 19 [38,39]. Therefore, HBA-enhanced abbreviated MRI may give important information on the spectrum of HCC progression. However, considering the increased cost and possible adverse effects of contrast media, the use of MRI as a primary surveillance modality in at-risk patients might be limited, but a strategy where MRI is used for patients who have both a high risk for HCC and are prone to US failure could be cost effective [25,40]. Therefore, our study should be clinically useful for determining those patients who would most benefit from an alternative surveillance modality to US.

Our study has several limitations. First, all six included studies were retrospective by design, causing a potential selection bias. Careful interpretation of our study would be needed, and future randomized studies are warranted. Second, substantial study heterogeneity was noted in the incidence of inadequate US examination among the included studies. To overcome this limitation, we robustly performed subgroup analyses according to various covariates. Third, the number of studies evaluating the risk factors for inadequate US examination was small (*n* = 5), leading to underpowered results.

## 5. Conclusions

In conclusion, 21.5% of US surveillance for detecting HCC in at-risk patients was found to be inadequate examination. High BMI (≥25 kg/m^2^), Child–Pugh B cirrhosis, and NASH were significant risk factors for inadequate US examination. Therefore, an alternative surveillance modality might be considered in patients who have any of these risk factors.

## Figures and Tables

**Table 1:** Characteristics of the included articles.

Author (Publication Year)	Study Design	Study Location	Cohort of Surveillance	No. of Patients (% Cirrhosis)	Age, Years *	Most Common Etiology	No. of Patients with HCC	US Machine	Sonographer Experience	Test for Surveillance	Interval of Test	Reference Standard	Follow-Up Period, Months *
Kim YY (2019)	Retrospective	South Korea	Patients with hepatic observation	155 (N.A.)	59.4 ± 8.9 *	Hepatitis B	155	NA	1–4 years	US plus AFP	6 months	Pathology, CT or MRI	NA
Millet JD (2019)	Retrospective	U.S.	Total cohort **	2050 (51.4)	57.7, mean	Hepatitis B	29	Multiple	19.3 ± 12.3 *	US plus AFP	6 months	Pathology, CT or MRI	21.7 ± 2.7 *
Pinero F (2019)	Retrospective	Argentina	Patients with hepatic observation	345 (100)	62 ± 8.8 * 61 ± 9.6 *	Hepatitis C	345	NA	NA	US plus AFP	6 months	Pathology, CT or MRI	NA
Son JH (2019)	Retrospective	South Korea	Total cohort **	407 (100)	56, median (28–76)	Hepatitis B	28	Single	>14 years	US alone	6 months	Pathology, CT or MRI	18, median
Samoylova ML (2018)	Retrospective	U.S.	Patients with hepatic observation	352 (NA)	60, median (56–65)	Hepatitis C	352	NA	NA	US plus AFP	NA	CT or MRI	NA
Simmons O (2017)	Retrospective	U.S.	Total cohort **	941 (100)	56.5 ± 9.9 *	Hepatitis C	NA	Multiple	NA	US plus AFP	6 months	NA	NA

Articles are listed according to the year of publication and alphabetical order of the names of the first authors within the same year of publication. * Data are presented as the means ± standard deviations. ** Total cohort includes patients without hepatic observations as well as those with hepatic observations. HCC, hepatocellular carcinoma; No, number; US, ultrasound; AFP, alpha-fetoprotein; CT, computed tomography; MRI, magnetic resonance imaging; NA, not available.

**Table 2:** Subgroup analysis for the incidence of inadequate US examination.

		Meta-Analytic Summary Estimate	
Covariates	Subgroup (Number of Study)	Pooled Incidence, % (95% CI)	*p*-Value
Surveillance cohort	Total cohort (*n* = 3) Patients with hepatic observations (*n* = 3)	23.2% (20.8–25.9) 18.8% (15.8–22.2)	0.03
Geographic difference	North America (*n* = 3) Others (*n* = 3)	20.8% (17.8–24.2) 22.6% (18.8–27.0)	0.48
Proportion of cirrhosis	All cirrhosis (*n* = 3) Not all cirrhosis (*n* = 3)	22.8% (19.3–26.7) 20.1% (16.6–24.1)	0.33
Surveillance test	US plus AFP (*n* = 5) US alone (*n* = 1)	20.8% (18.8–23.0) 28.0% (23.1–33.5)	0.008
US system	Multiple systems (*n* = 2) Single system (*n* = 4)	21.8% (18.0–26.1) 21.2% (17.9–25.0)	0.84
Reference standard	Pathologic and imaging diagnosis (*n* = 4) Imaging diagnosis only (*n* = 1)	23.2% (21.8–24.8) 17.9% (14.2–22.3)	0.02

CI, confidence interval; US, ultrasound; AFP, alpha-fetoprotein.

**Table 3:** Pooled odds ratios of the risk factors for inadequate US examination.

	Meta-Analytic Summary Estimate		
Risk Factor	Pooled Odds Ratio (95% CI)	*I*^2^ Statistics	*p*-Value
BMI (≥25 kg/m^2^)	2.2 (1.12–4.24)	58%	0.03
Age (≥60 years)	1.1 (0.08–14.25)	0%	0.76
Male	1.5 (0.90–2.38)	0%	0.08
Child-Pugh classification (B) *	2.2 (1.10–4.37)	0%	0.04
Hepatitis B	1.1 (0.41–2.93)	8%	0.73
NASH	2.3 (1.07–4.84)	33%	0.04
Alcohol-related	1.7 (0.06–44.91)	43%	0.29
ALT > 40 (U/L)	1.0 (0.11–8.96)	0%	1.00
MELD score (≥11)	1.9 (0.02–179.92)	73%	0.32

* Compared with Child–Pugh classification A. US, ultrasound; CI, confidence interval; BMI, body mass index; NASH, nonalcoholic steatohepatitis; ALT, alanine aminotransferase; MELD, model for end-stage liver disease.

## Data Availability

All data accessed are available in the article and its Supplementary Materials.

## Supplementary Materials

The following are available online at https://www.mdpi.com/article/10.3390/jcm10163535/s1, Figure S1: Forest plots of the odds ratios of age (A), sex (B), hepatitis B infection (C), alcohol abuse (D), alanine aminotransferase (ALT) (E), and model for end-stage liver disease (MELD).(F), Figure S2: Funnel plot for examination of publication bias in studies reporting results for the rate of inadequate US examinations, Table S1: Search queries, Table S2: Newcastle-Ottawa Scale for assessment of the risk of bias in the included studies.
