# Supplementary material for: Inadequate Ultrasound Examination in Hepatocellular Carcinoma Surveillance: A Systematic Review and Meta-Analysis
Source: J Clin Med. 2021 Aug 12;10(16):3535. doi: 10.3390/jcm10163535 (PMC8397222; doi:10.3390/jcm10163535)
Supplement: Supplementary file 1 [file jcm-10-03535-s001.zip › jcm-1330568-supplementary.pdf]

Supplementary Table S1. Search queries

No.	Search queries for MEDLINE
#1	"Carcinoma, Hepatocellular"[Mesh] OR "Liver Neoplasms"[Mesh:NoExp]
#2	(liver*[TW] OR hepatic*[TW] OR Hepato*[TW]) AND (carcinoma*[TW] OR cancer[TW] OR cancers[TW] OR tumor[TW] OR neoplas*[TW] OR malignan*[TW])
#3	hepatocarcinoma*[TW] OR hepatoma*[TW] OR "liver carcinoma"[TW] OR HCC[TW]
#4	#1 OR #2 OR #3
#5	"Ultrasonography"[Mesh]
#6	ultrasonograp*[TW] OR Ultrasound*[TW] OR Ultrasonic*[TW]
#7	#5 OR #6
#8	surveillanc*[TW] OR Monitoring*[TW]
#9	"Population Surveillance"[Mesh] OR "Early Detection of Cancer"[Mesh] OR "mass screening"[MeSH] OR "Epidemiological Monitoring"[Mesh]
#10	#8 OR #9
#11	"Predictive Value of Tests"[Mesh] OR "Sensitivity and Specificity"[Mesh]
#12	predictive value*[TW] OR predictor*[TW] OR detection rate*[TW] OR "False Negative"[TW] OR "False positive"[TW] OR "True Negative"[TW] OR "True positive"[TW] OR "PPV"[TW] OR "NPV"[TW] OR OR Sensitivit*[TW] OR Specificit*[TW] OR accurac*[TW]
#13	failure*[TW] OR adequat*[TW] OR inadequat*[TW] OR poor*[TW]
#14	#11 OR #12 OR #13
#15	#4 AND #7 AND #10 AND #14
#16	#15 AND (English[Lang])
No.	Search queries for EMBASE
#1	'liver cell carcinoma'/exp OR 'liver cancer'/de
#2	((liver* OR hepatic* OR Hepato*) NEAR/6 (Carcinoma* OR Cancer* OR tumor* OR Neoplas* OR malignan*)):ab,ti,kw
#3	(hepatocarcinoma* OR hepatoma* OR 'liver carcinoma' OR HCC):ab,ti,kw

#4	#1 OR #2 OR #3
#5	'echography'/exp
#6	(ultrasonograp* OR Ultrasound* OR Ultrasonic*):ab,ti,kw
#7	#5 OR #6
#8	(surveillanc* OR Monitoring*):ab,ti,kw
#9	'early cancer diagnosis'/exp OR 'cancer screening'/exp OR 'disease surveillance'/exp
#10	#8 OR #9
#11	'diagnostic accuracy'/exp OR 'predictive value'/exp OR 'sensitivity and specificity'/de
#12	('predictive value' OR 'predictive values' OR predictor* OR 'detection rate' OR 'detection rates' OR 'False Negative' OR 'False positive' OR 'True Negative' OR 'True positive' OR 'PPV' OR 'NPV' OR Sensitivit* OR Specificit*):ab,ti,kw
#13	(Diagnos* NEAR/3 Accurac*):ab,ti,kw
#14	(failure* OR adequat* OR inadequat* OR poor*):ab,ti,kw
#15	#11 OR #12 OR #13 OR #14
#16	#4 AND #7 AND #10 AND #15
#17	#16 AND ([english]/lim)
No.	Search Queries for Cochrane
#1	[mh "Carcinoma, Hepatocellular"] or [mh ^"Liver Neoplasms"]
#2	((Hepatocellular* or liver-cell* or hepatic-cell* or Hepato-cell*) near/6 (Cancer* or tumor* or Neoplas* or carcinoma*)):ab,ti,kw
#3	hepatocarcinoma*:ab,ti,kw or hepatoma*:ab,ti,kw or "liver carcinoma":ab,ti,kw or HCC:ab,ti,kw
#4	#1 or #2 or #3
#5	[mh "Ultrasonography"]
#6	(ultrasonograp* or Ultrasound* OR Ultrasonic* OR US):ab,ti,kw
#7	#5 or #6
#8	surveillanc*:ab,ti,kw or Monitoring*:ab,ti,kw
#9	[mh "Population Surveillance"] or [mh "Early Detection of Cancer"] or [mh "mass screening"] or [mh "Epidemiological Monitoring"]

#10	#8 or #9
#11	[mh "Predictive Value of Tests"] or [mh "Sensitivity and Specificity"]
#12	(predictive value* or predictor* or detection rate* or "False Negative" or "False positive" or "True Negative" or "True positive" or "PPV" or "NPV" or Sensitivit* or Specificit* or accurac*):ab,ti,kw
#13	[mh "Risk Factors"] or (Risk-Factor* or failure* or adequat* or inadequat* or poor*):ab,ti,kw
#14	#11 or #12 or #13
#15	#4 and #7 and #10 and #14
#16	#15 in Trials(Published)

Supplementary Table S2. Newcastle-Ottawa Scale for assessment of the risk of bias in the included studies

	Selection				Comparability	Outcome			Total
Author (year)	Representativeness of the exposed cohort	Selection of the non-exposed cohort	Ascertainment of exposure	Demonstration that outcome of interest was not present at start of study	Comparability of cohorts on the basis of the design or analysis	Assessment of outcome	Was follow-up long enough for outcomes to occur	Adequacy of follow up of cohorts	
Simmons O (2017)	1	1	1	1	2	1	0	0	7
Samoylova ML (2018)	1	1	1	1	1	0	0	0	5
Kim YY (2019)	1	1	1	1	2	1	0	0	7
Millet JD (2019)	1	1	1	1	2	1	1	0	8
Son JH (2019)	1	1	1	1	2	1	1	1	9
Pinero F (2019)	1	1	1	1	0	0	0	0	4

Articles are listed according to year of publication and alphabetical order of the names of the first authors within the same year of publication.

Supplementary Figure S1. Forest plots of the odds ratios of age (A), sex (B), hepatitis B infection (C), alcohol abuse (D), alanine aminotransferase (ALT) (E), and model for end-stage liver disease (MELD) (F).

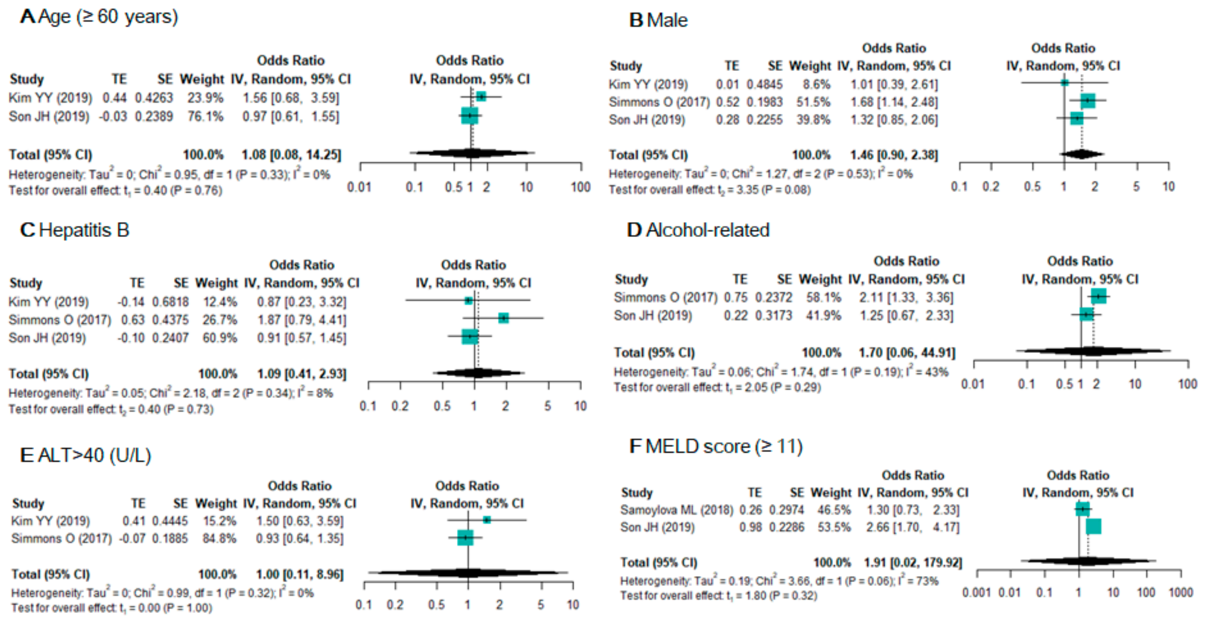

Supplementary Figure S2. Funnel plot for examination of publication bias in studies reporting results for the rate of inadequate US examinations

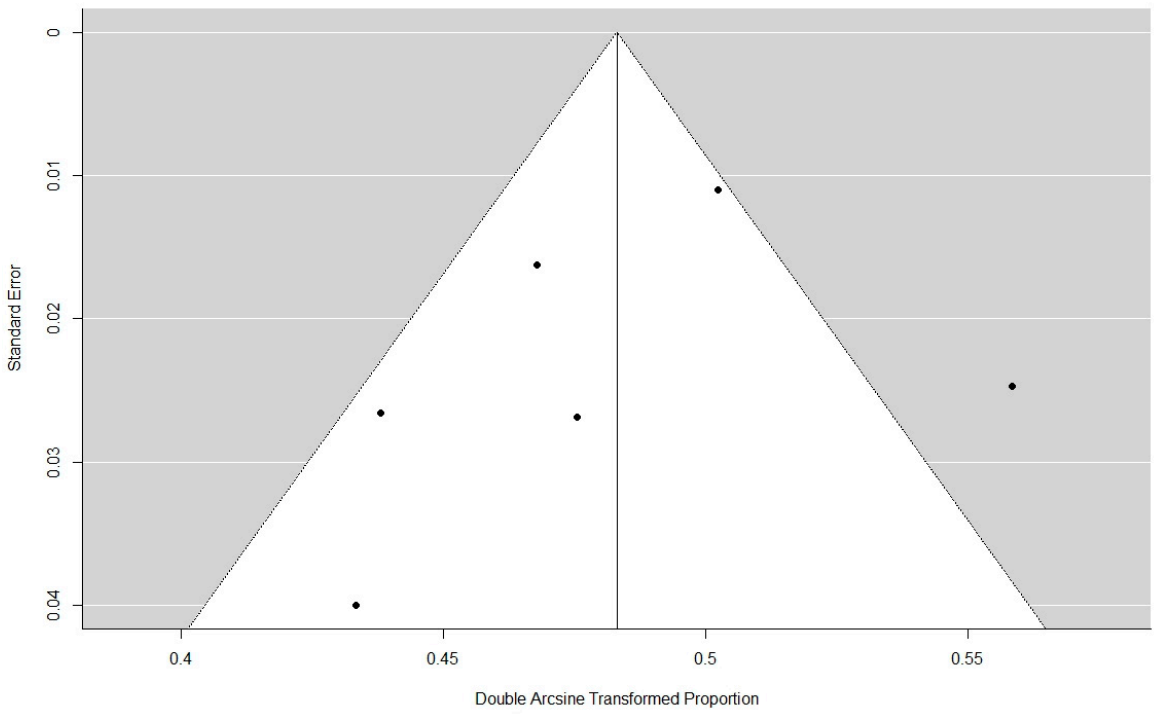

Standard Error
0
0.01
0.02
0.03
0.04
0.4
0.45
0.5
0.55
Double Arcsine Transformed Proportion
